# Materia Medica and Therapeutics

**Published:** 1859-01

**Authors:** 


### MATERIA MEDICA AND THERAPEUTICS.

1. TARTAR EMETIC IN LARGE DOSES IN THE TREATMENT OF CHOREA. By *M. Gillette.*—In the session of March 6th, 1858, of the "Société Médicale d'Emulation," M. Gallard mentioned a thesis of M. Bonfils on the use of tartar emetic in large doses in the treatment of several cases of chorea. As the facts reported in the essay of M. Bonfils had been observed in the service of M. Gillette, the latter was requested to explain to the Society his mode of treatment. It is the following :—

For the first day he prescribes ten centigrammes of tartar emetic, which the patient takes from hour to hour. In the course of the day vomiting generally supervenes ; if it becomes too frequent, the medicine is given less frequently, or is entirely suspended. On the second day he prescribes twenty-five centigrammes ; this produces, perhaps, some vomiting still. On the third day, thirty centigrammes ; this is generally *not* attended by vomiting or purging. After this period, three or four days rest. There is already an improvement, and a perceptible amelioration of the disease. In exceptional cases, the cure is obtained thus early. The patient is subjected to the same treatment for another period of three days, during which M. Gillette prescribes progressively, twenty-five, fifty, and seventy-five centigrammes of tartar emetic. This period is also followed by three or four days of repose, after which the dose of the medicine is pushed to thirty, sixty, and one hundred grains. The improvement is such, that no disorderly movements are any more perceived. The case may then be confirmed by ordinary means, particularly gymnastics and sulphur-baths; but this is a precaution dictated more by custom than by necessity.

Since the thesis of M. Bonfils, several new facts of cure have been observed. The use of tartar emetic in the treatment of chorea has afforded to M. Gillette actually thirty-seven cures and only one failure in thirty-eight patients submitted to his observation. The author's attention in the administration of the medicine is particularly directed toward establishing, as soon as possible, a tolerance of the remedy ; perturbation of the system, by producing the violent physiological effects of the emetic, should be avoided. The chorea disappeared progressively, and the sooner, the more intense the affection had been.

M. Brienne de Boismont reports a case cured in five days by the method of M. Gillette.

There was nothing said of the local action of tartar emetic on the mucous membrane, and it is well known that the prolonged influence of the medicine has some inconveniences.—*Union Médicale,* June 12, 1858.

2. ON THE USE OF PROPYLAMIN IN RHEUMATISM. By *Dr. Amenarius.*—Propylamin, a peculiar volatile alkali, obtained from herring-pickle, cod-liver oil, ergot, human urine, etc. promises, according to the observations of Dr. Amenarius, to become a real specific in the different diseases which are of purely *rheumatic* origin; nay, in cases where the diagnosis is doubtful, the exhibition of propylamin, continued for a few days, will even afford a criterion of the true nature of the malady. The author used this remedy, with excellent results, in two hundred and thirty cases of acute and chronic rheumatism, which came under his observation within a period of three years, in the Kalinkin Hospital, in St. Petersburg, Russia, and treated a large number of patients outside the hospital with equal success. Most cases were: partial or general muscular rheumatism, rheumatic facial neuralgia, metastatic rheumatism of the pericardium, membranes of the brain, or pleura, hemiplegia and paraplegia of the lower extremities. In acute cases, pain and fever had often disappeared on the next day. Form and dose:—℞. Propylamin, gtt. xxiv in aq. distil. ℥vi, to which may be added Elæosacchar. Menth. pisst. ʒij; every two hours a tablespoonful. —*Med. Ztg.*, Russl. 6, 1858; and *Schmidt's Jahrbücher*, 7, 1858.

3. ON THE EXTERNAL USE OF HYDROCHLORIC ACID. By *Prof. V. Kletpinsky.* —In the course of some investigations made with reference to the physiology and pathology of the skin, Prof. Kletpinsky found that, among all the agents subjected to the test, none was as efficient in exciting the respiratory function of the skin, accelerating the capillary circulation, and influencing the action of the lymphatic and glandular system, as hydrochloric acid. Skin moistened with hydrochloric acid expired, under the same circumstances, twenty-seven to thirty per cent. more of carbonic acid, and, what is very remarkable, seven to twelve per cent. less of water, than an equal space of skin not subjected to the influence of the acid. This fact induced the author to apply the acid in a great number and variety of cases, in order to test its therapeutical efficacy. He obtained the following results:—

1. Hydrochloric acid restores and stimulates the circulation if periodically interrupted or stagnating; it thus cures frost-bite and chilblain, and is an efficient prophylactic against these complaints.

2. The acid diminishes the troublesome perspiration of the hands and feet, and cures it in some cases completely, if the application is continued long enough.

3. It is an efficient remedy in a great variety of cutaneous diseases, particularly in follicular acne; by rendering the metamorphosis of tissues more active, it destroys, if steadily used, many maculæ and exudative patches of the skin.

4. It does not injure the integrity of the epidermis, if properly applied, but diminishes its roughness and callosities; like a true cosmetic, it renders the skin pliable and soft, increasing at the same time its density, and making it, consequently, more resistant to obnoxious influences.

The hydrochloric acid, which must be free from admixture of iron or chlorine, is best applied in as concentrated a condition as can be borne, without its giving rise to burning; commonly, a more concentrated acid can soon be made use of, even if it was inapplicable at the beginning of the treatment. The skin is moistened with the acid, (which was used by the author in many cases even in its fuming state,) and is washed off after a quarter of a minute, at first with water and then with soap. It is easily understood, that the hands bear most easily the concentrated acid, the feet (especially the toes) less, the forehead the least; on all sensitive places of the skin the acid must be applied more diluted and for a shorter time. It is an excellent plan to mix the hydrochloric acid with glycerin, the therapeutical action of which on the skin is hardly enough appreciated, and which in this case renders a longer application of the acid, even to a sensitive skin, practicable.—*Oesterreichische Zeitschrift für praktische Heilkunde*, No. xii.

4. ERGOTIN IN PULMONARY TUBERCULOSIS.—*Dr. G. Ferrini* recommends ergotin as an efficient remedy in pulmonary tuberculosis, as it not only diminishes the cough, but also reduces the congestion of the lungs, and by thus checking the progress of the disease, acts not merely in a palliative, but in a truly curative manner. The author gives the remedy in doses of fifteen to twenty grains daily in mucilage, and aids the treatment by the use of counter-irritants, and depletion, if necessary. Dr. Ferrini supports his recommendation by a great number of cases, of which he communicates five more fully.—*Annali Univ.*, Agosto, Settembre, 1857.

5. ACETATE OF COPPER IN PRURITIS. By *Dr. G. V. Lafargue.*—The author communicates two cases of chronic hyperæsthesia of the skin, accompanied by violent itching, (no anatomical changes being perceptible,) in which he used a solution of ten centigrammes of neutral acetate of copper in twenty tablespoonfuls of water, (mornings and evenings, a tablespoonful—five milligrammes of the salt; later, twelve milligrammes.) After a few weeks a complete cure was obtained. Derangement of the digestive organs was not observed.—*Bull. de Thér.* liv., p. 168; 1858.

6. NITRATE OF SILVER IN THE TREATMENT OF OZÆNA.—*Dr. Gallizioli* recommends, in the *Raccoglitore Medico di Fano*, the use of nitrate of silver in the treatment of ozæna. The salt is either snuffed up, mixed with the powder of the iris florentina, or introduced into the nostrils in the form of ointment. In evidence of the usefulness of this method he reports several cases, of which we present only the following:—

A young girl was cured of ecthyma by the use of sulphur-baths and laxatives; she suffered, however, at the same time, from ozæna, which had distressed her and her family for eight years on account of its repulsive smell. Dr. Gallizioli ordered an ointment of 40 centigrammes argent. nitrat. to 30 grammes axung. porc.; pledgets of charpie were smeared with this and introduced deep into the nostrils. The application was renewed every day, and in sixteen days the patient was completely cured.

7. RESEARCHES ON THE THERAPEUTICAL ACTION OF THE PERCHLORIDE OF IRON IN THE TREATMENT OF ACUTE AND CHRONIC URETHRITIS. By *M. Barudel.*—It results from the researches of M. Barudel, that the perchloride of iron, besides its hæmostatic properties, for which it is so often applied, possesses, on internal administration, a very manifest sedative action on the general circulation. In thirty patients subjected to this treatment the pulse fell in two or three days from seventy or eighty a minute to sixty and even fifty; this salt produces neither cramps, pinching, or twinging of the stomach, nor uneasiness in the cardiac region, colic, nor constipation. The chloride of iron has been administered by M. Barudel with remarkable results both in acute and chronic urethritis. In the acute form he orders an injection of 10 grammes of the iodide of lead in 100 grammes of water to be used three times daily, and administers at the same time the following potion:—

℞.—Distilled water, 60 grammes;
Perchloride of iron of 30°, 20 drops;
Simple syrup, 15 grammes.

To be taken every two hours. Continue this potion for ten days.

In the chronic form, the internal treatment is exactly the same; for the injection of the iodide of lead the following is substituted:—

℞.—Perchloride of iron of 30°, 25 drops;
Distilled water, 100 grammes.

Inject three times daily, taking care to keep the liquid for ten minutes in the urethra.

If the pain provoked by this injection is too severe and lasts too long, it should be followed by two or three injections of cold water, and a day of repose should be permitted to the patient. This treatment has never given rise to any accident. It produced generally a marked improvement in three days, and often a cure in five days. M. Barudel combines with it a tonic regimen and cooling drinks, such as milk and flaxseed tea with nitre.

These results, and the two observations chosen from a great number of others, with which M. Barudel concludes his article, should induce physicians to try the proposed treatment.—*Bulletin Général de Thérapeutique*, May 15, 1858.

